# Supplementary material for: Association between oral microbiome and seven types of cancers in East Asian population: a two-sample Mendelian randomization analysis
Source: Front Mol Biosci. 2023 Nov 21;10:1327893. doi: 10.3389/fmolb.2023.1327893 (PMC10702768; doi:10.3389/fmolb.2023.1327893)
Supplement: Supplementary file 1 [file DataSheet1.ZIP › Supplementary Materials/MR plots for tongue/tongue═╝/Pancreatic cancer/╥╚╧┘░⌐_╔╕╤í╜ß╣√/pheno.3074_to_pancreatic cancer_forest.pdf]

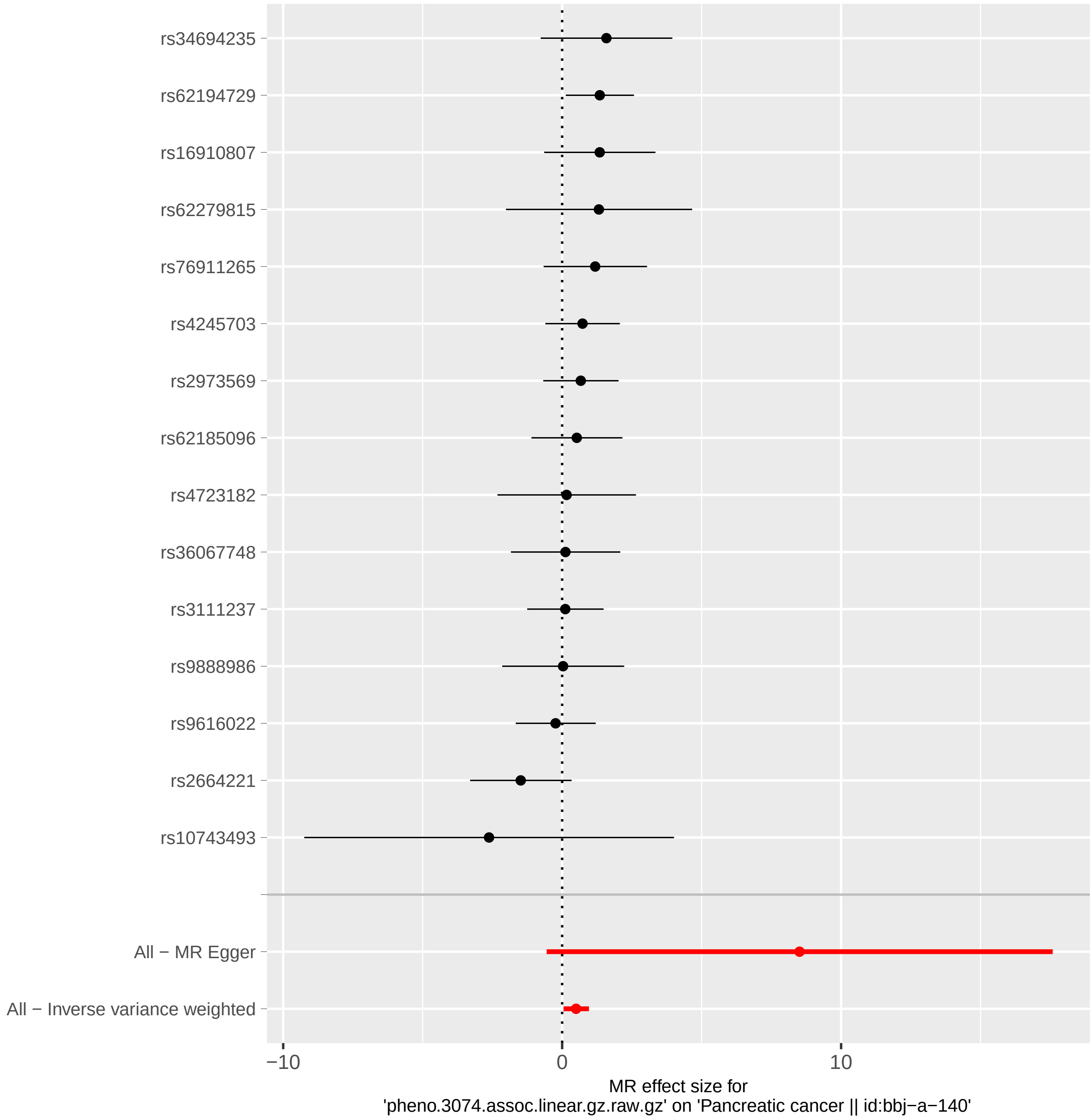

rs34694235
rs62194729
rs16910807
rs62279815
rs76911265
rs4245703
rs2973569
rs62185096
rs4723182
rs36067748
rs3111237
rs9888986
rs9616022
rs2664221
rs10743493
All − MR Egger
All − Inverse variance weighted
−10
0
10
MR effect size for
'pheno.3074.assoc.linear.gz.raw.gz' on 'Pancreatic cancer || id:bbj−a−140'
